# Assessing metrics for evaluating the maternal continuum of care in Ethiopia: A scoping review

**DOI:** 10.1371/journal.pgph.0007303

**Published:** 2026-09-15

**Authors:** Abdulaziz Mohammed Hussen, Ibrahim Mohammed Ibrahim, Martin Heine, Tigest Tamrat, Özge Tunçalp, Binyam Tilahun, Diederick E. Grobbee, Joyce L. Browne

**Affiliations:** 1 Department of Global Public Health & Bioethics, Julius Centre for Health Sciences and Primary Care, University Medical Centre Utrecht, Utrecht University, Utrecht, Netherlands; 2 Department of Midwifery, College of Medicine and Health Science, Samara University, Samara, Ethiopia; 3 Center for Digital Health and Implementation Sciences (CDHI), University of Gondar, Gondar, Ethiopia; 4 Department of Exercise, Sport and Lifestyle Medicine, Faculty of Medicine and Health Sciences, Institute of Sport and Exercise Medicine, Stellenbosch University, Stellenbosch, South Africa; 5 Department of Sexual and Reproductive Health and Research, UNDP—UNFPA—UNICEF—WHO—World Bank Special Programme of Research, Development and Research Training in Human Reproduction (HRP), World Health Organization, Geneva, Switzerland; 6 Institute of Tropical Medicine, Antwerp, Belgium; 7 Department of Health Informatics, Institute of Public Health, College of Medicine and Health Sciences, University of Gondar, Gondar, Ethiopia; African Population and Health Research Center, KENYA

## Abstract

Despite Ethiopia’s remarkable progress in reducing maternal mortality between 2000 and 2023, more effort is needed to achieve the Sustainable Development Goal (SDG) target. The government outlined a continuum of care framework for reproductive, maternal, and child healthcare service delivery to positively impact maternal health outcomes. Therefore, it is important to measure and monitor progress in improving women’s access to the continuum of care. However, there is no standardized metric to evaluate whether women receive comprehensive, coordinated care throughout pregnancy, childbirth, and the postpartum period. This absence makes it difficult to assess service integration, identify gaps in care transitions, and inform policy decisions aimed at strengthening the maternal healthcare system. This review aimed to assess the maternal continuum of care measurement metrics. The review used the Joanna Briggs Institute (JBI) scoping review framework. Relevant studies were searched on MEDLINE (via PubMed), Scopus, Web of Science (WOS), and Google Scholar. Reference lists of the included articles were also reviewed for additional studies. Screening was carried out by two reviewers, and disagreements were resolved through consensus. Definitions of the maternal continuum of care were extracted from the included studies. Moreover, the definitions were compared with national maternal healthcare service recommendations. A total of 496 studies were identified, of which 29 were included. All definitions of the ‘maternal continuum of care’ focused on coverage of maternal healthcare services across three periods: pregnancy, childbirth, and postpartum. Few studies (n = 2) defined the continuum of care in line with the national maternal healthcare service recommendations. Our findings indicate a need for a consistent, holistic measurement metric that addresses both coverage and quality of services and is aligned with maternal healthcare service recommendations. By introducing a new standardized metric, efforts to improve the continuum of care will be strengthened with the necessary data to track progress and guide evidence-based interventions, ensuring a more integrated and effective maternal healthcare system. The development of this metric should leverage existing frameworks for assessing the quality of maternal healthcare services.

## Introduction

Most (70%) of maternal deaths globally occur in Sub-Saharan Africa [1]. In this region, the probability that a 15-year-old girl will die from complications of pregnancy or childbirth over her lifetime was estimated (in 2023) to be 1 in 55. This risk is about five times higher than the global average (1 in 272) and more than 380 times higher than in high-income country settings such as Australia and New Zealand (1 in 21,248) [1]. Sustainable Development Goal (SDG) 3’s target expresses the ambition to reduce the global Maternal Mortality Ratio (MMR) to less than 70 per 100,000 live births by 2030, and that no country should have maternal mortality more than double the global target [1,2]. If the 2016–2023 pace of reduction continues, the global MMR will be 177 per 100,000 live births by 2030, two and a half times higher than the SDG target. To meet the target and prevent an estimated 700,000 maternal deaths, an average Annual Rate of Reduction (ARR) of 14.8% is required over the remaining seven years (2024–2030) [1]. To achieve that, a significant rise in maternal healthcare utilisation before, during, and after pregnancy, as well as an improvement in the quality of the services, is necessary [2–4].

The continuum of care for maternal, neonatal, and child health has become a rallying call to reduce preventable maternal and neonatal deaths [5]. This continuum has been broadly defined over two dimensions: the dimension of time (throughout the lifecycle), and the dimension of place or level of care. The time dimension includes care before pregnancy (including family planning services and preconception care), during pregnancy, childbirth, and the postnatal period. The dimension of place or level includes the care provided at home, primary health care facilities, and higher‑level hospitals [5]. There are recommended services a woman should receive during prenatal, childbirth, and postpartum periods [6–11], and continuous utilisation is associated with a lower risk of obstetric problems and unfavourable birth outcomes [12,13].

Despite Ethiopia’s remarkable progress in reducing maternal mortality between 2000 and 2023, from 870 per 100,000 live births in 2000–195 per 100,000 live births in 2023 [1], the mortality is still far from the SDG target [2]. Moreover, Ethiopia ranked fifth among countries with the highest maternal deaths in 2023, with 8,000 deaths, accounting for 3.1% of all estimated global maternal deaths [1]. The Ministry of Health (MOH) of Ethiopia, in its national reproductive health strategy [14], outlined a continuum of care framework for reproductive, maternal, and child healthcare service delivery. This framework emphasizes continuity across two dimensions: time (connecting essential reproductive health packages throughout adolescence, pregnancy, childbirth, postpartum, and childhood, building upon their natural interactions throughout life) and place (strengthening links between households, first-level facilities, and hospitals, bringing care closer to the home through outreach services and promoting referral to higher‑level facilities). Moreover, the continuum of care is highlighted as a key maternal health service delivery approach in national health strategy documents [14–16].

Effective care along the continuum is expected to have a positive impact on maternal health outcomes [5,16]. Therefore, measuring and monitoring progress in improving women’s access to the continuum of care is essential. Despite the availability of service-specific recommendations, for example, the recommended services during antenatal care (ANC), in national guidelines and strategic documents [10,11,15,16], there is no standardized metric to evaluate whether women receive comprehensive, coordinated care throughout pregnancy, childbirth, and the postpartum period. This absence makes it difficult to assess service integration, identify gaps in care transitions, and inform policy decisions aimed at strengthening the maternal healthcare system. Without well-defined metrics, efforts to improve the continuum of care may lack the necessary data to track progress and guide evidence-based interventions. This review aimed to assess maternal continuum of care measurement metrics. Understanding the current measurement metrics and highlighting potential areas for improvement will guide and contribute to monitoring efforts and future research in this area.

## Methods

The scoping review was conducted by following the Joanna Briggs Institute (JBI) scoping review framework [17], and it’s reported according to the Preferred Reporting Items for Systematic Reviews and Meta-Analyses extension for Scoping Reviews (PRISMA-ScR) guideline (S1 Checklist).

### Eligibility criteria

All types of primary studies published in English at any time were eligible for inclusion if they captured the following Population, Concept, and Context (PCC) domains [17].

### Population

N/A

### Concept

The concept in this review is the continuum of care for maternal health services. Studies that measured the maternal continuum of care were considered.

### Context

Studies conducted in Ethiopia.

**Information sources:** An electronic search was conducted from October 27–29, 2023, across four databases: MEDLINE (via PubMed), Scopus, Web of Science (WoS), and Google Scholar.

**Selection of sources of evidence:** A 3-step search strategy was followed. An initial search of MEDLINE (via PubMed) and Scopus databases for relevant articles was followed by an analysis of text words in the title and abstract and index terms used to describe the article. Then, a second search was carried out using all the identified MeSH terms (PubMed) and keywords (all databases) across all databases, PubMed, Scopus, WoS, and Google Scholar. Finally, the reference list of the selected/included articles was searched for additional studies (S1 Text). Studies retrieved from the search were exported to reference management software (Mendeley and EndNote). After removing duplicate results, two reviewers (AMH and IMI) screened all titles and abstracts independently to identify relevant studies for full text, and the full texts of the selected abstracts were reviewed. Disagreements between the reviewers were resolved through consensus. AMH used Mendeley and IMI used EndNote. A study was selected for the review when the full text fulfilled the eligibility criteria.

### Search strategy for PubMed

((“Continuity of Patient Care”[Mesh] OR “Continuum” [tiab] OR “Continuum of Care” [tiab] OR “Care Continuum” [tiab] OR Continu*[tiab]) AND (“Maternal Health Services”[Mesh] OR “Maternal Health service” [tiab] OR “Maternity care” [tiab] OR “Maternity servic*”[tiab] OR “Obstetrics servic*”[tiab])) AND (“Ethiopia”[Mesh] or Ethiopia [tiab])

### Data extraction and analysis

The characteristics of the included studies were summarized using a template that was aligned with the review objective. The template contains categories for descriptive characteristics of the included studies, such as Author/s, year of publication, and the definition of the continuity of care for maternal health services. All definitions were narratively synthesized into a single table, and frequencies were produced to compare the definitions. To assess alignment, study definitions were compared with Ethiopian national maternal health service recommendations.

In Ethiopia, pregnant women are expected to have frequent contact with healthcare providers and receive recommended care across antenatal, childbirth, and postpartum periods. The latest (2022) national ANC guideline recommends eight antenatal contacts [11], before that, four ANC visits were the recommended number of ANC visits [18]. For childbirth, the Health Sector Transformation Plan (HSTP) of Ethiopia states “All pregnant women are encouraged and supported to deliver in health facilities with skilled attendants” [19]. Despite the above recommendation in the HSTP document, we noted the use of “institutional delivery” and “skilled birth attendant” terms interchangeably in policy and strategy documents [14–16,19,20]. Moreover, at least four postnatal contacts, within 24 hours, at day 3 (48–72 hours), between days 6th and 7th, and at 6 weeks after delivery, are recommended [10]. In addition to the number of visits, the recommendations also cover the type of services that should be provided during each contact.

## Results

### Description of the studies

**Fig 1:**
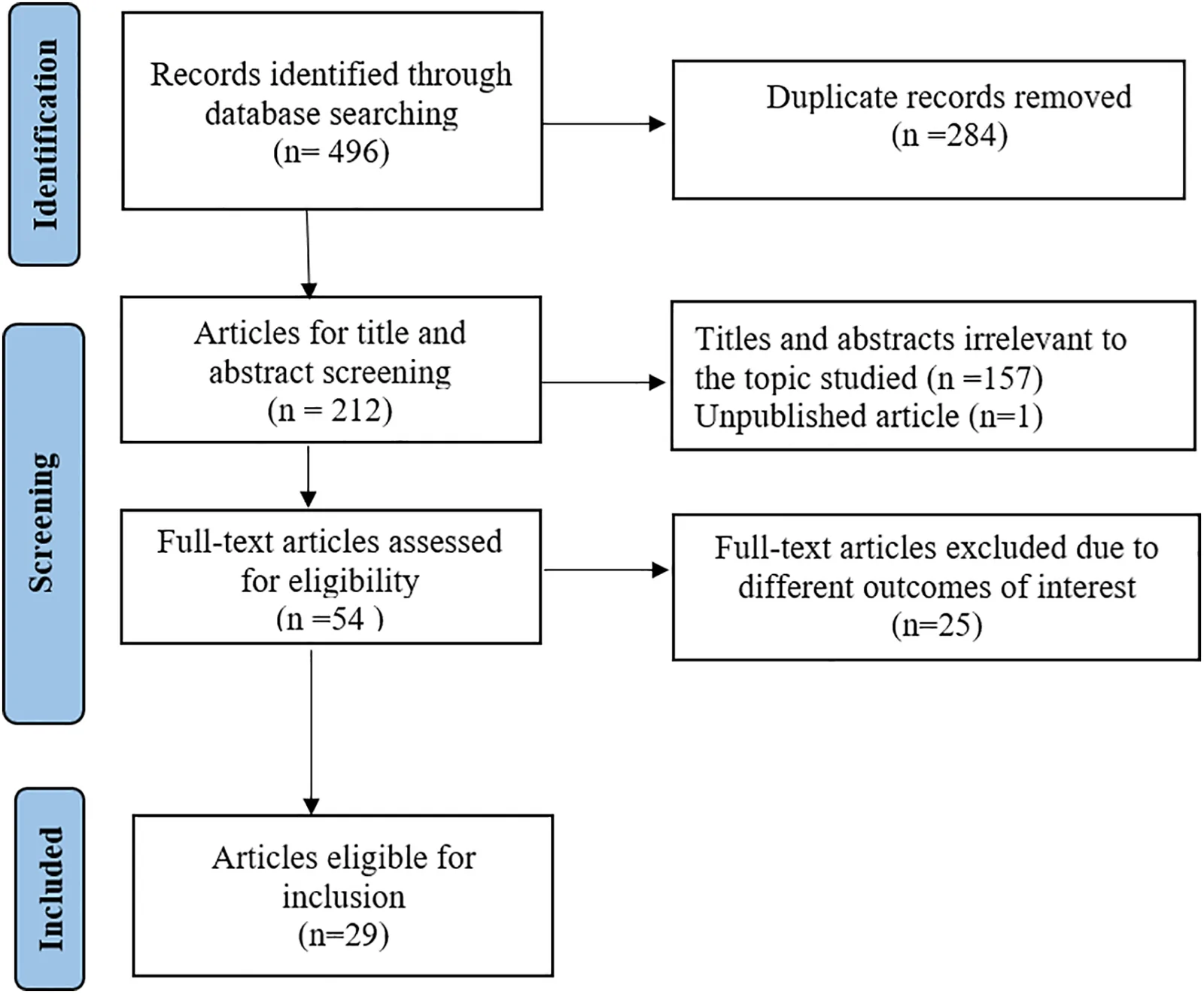
PRISMA flow chart: overview of article screening and selection process.

The systematic search resulted in 496 articles, of which 212 remained after removing duplicates. After screening, 29 articles were included in this scoping review (Fig 1). The year of publication of the included studies was between 2019 and2023, and all the studies were quantitative in type (Table 1).

**Table 1:** Definitions of the maternal continuum of care in published studies and their alignment with national maternal health service recommendations (N = 29).

Author (s)	Year of publication	Definition of the continuity of maternity care	Alignment with national maternal healthcare service recommendations		
			Pregnancy*	Childbirth**	Post partum*
Chaka E.E. et al [21]	2019	At least four ANC visits, SBA, and PNC			
Asratie M.H. et al [22]	2020	At least one ANC, childbirth aided by SBA (doctor, nurse, and midwife, health officer, and health extension worker), and at least one PNC within 6 weeks			
Atnafu A et al [23]	2020	At least four ANC visits, Childbirth at home or a health facility by a trained health professional, and PNC within 48 hours			
Emiru A.A et al [24]	2020	At least four ANC visits, skilled delivery, and PNC within 48 hours			
Haile D et al [25]	2020	At least four ANC visits, childbirth at a health facility, and PNC within 6 weeks (at least once after discharge from health facilities or within the first week after childbirth at their home)			
Muluneh A.G. et al [26]	2020	At least four ANC visits, skilled delivery, and at least one PNC			
Shitie A et al [27]	2020	At least one ANC visit, childbirth aided by SBA, and PNC within 48 hours			
Cherie N et al [28]	2021	At least four ANC visits, childbirth aided by SBA, and three PNC at a health facility or at home, within 24 hours, within 3 days, and within a week.			
Dadi T.L. et al [29]	2021	At least four ANC, institutional delivery, and PNC			
Sertsewold S.G. et al [30]	2021	At least four ANC visits, skilled delivery, and at least one PNC within 6 weeks by a skilled health worker			
Tizazu M.A. et al [31]	2021	At least one ANC visit, childbirth by skilled health personnel (medical doctors, midwives, nurses, health officers, or community health extension workers), and at least one PNC after discharge from the health facility within six weeks by skilled health personnel (medical doctors, midwives, nurses, health officers, or community health extension workers) or with in the first week by community health extension workers during their home visit.			
Abebe G.F. et al [32]	2022	At least four ANC visits, institutional delivery, and PNC service after delivery			
Ahmed R et al [33]	2022	At least four ANC visits, facility delivery (SBA), and PNC within 48 hours			
Amare N.S. et al [34]	2022	At least one ANC, SBA at a health institution, and a revisit to the facility to receive PNC			
Chaka E.E. et al [35]	2022	At least four ANC visits, SBA (delivery attended by doctor, nurse/midwife or health officer), and at least one PNC at a health facility or home.			
Hailemariam T et al [36]	2022	At least four ANC visits, SBA, and PNC within 48 hours			
Melaku M.S. et al [37]	2022	At least four ANC visits, delivery assisted by SBA, and PNC within 48 hours			
Tadese M et al [38]	2022	At least one ANC visit, SBA (delivery assisted by midwives, nurses, doctors, and/or health officers), and PNC within 6 weeks			
Tenaw S.G. et al [39]	2022	At least one ANC visit, delivered at a health institution assisted by SBA (doctors, midwives or nurses), and at least one PNC within 42 days			
Tiruneh G.T. et al [40]	2022	At least one ANC visit by health service providers at a health facility or home, delivery assisted by a health professional (i.e., doctor, nurse, midwife, or health officer/), and at least one PNC within 6 weeks by health service providers at a health facility or home			
Tsega D et al [41]	2022	At least one ANC visit, delivery at a health institution assisted by SBA (doctor, midwife, or nurse in the health institution), and at least one PNC within 42 days			
Zelka M.A. et al [42]	2022	Utilization of 1^st^ ANC, 2^nd^ ANC, 3^rd^ ANC, and 4^th^ ANC, Skill delivery care, 1^st^ PNC, 2^nd^ PNC, 3^rd^ PNC and 4^th^ PNC services			
Zelka M.A. et al [43]	2022	Utilization of 1^st^ ANC, 2^nd^ ANC, 3^rd^ ANC, and 4^th^ ANC, childbirth at a health facility or attended by a skilled provider, 1^st^ PNC, 2^nd^ PNC, 3^rd^ PNC and 4^th^ PNC services			
Alemayehu M et al [44]	2023	At least one ANC visit, delivery at a health facility and had PNC within 42 days			
Birhanu F et al [45]	2023	At least four ANC visits, childbirth at a health facility, and PNC within 48 hours			
Buli T.D. et al [46]	2023	At least four ANC visits, childbirth attended by an SBA (a medical doctor, midwife, nurse, and healthOfficer), and PNC within the first week after delivery at a health facility [excluding pre-discharge postnatal care] or home by a skilled provider			
Hussen A.M. et al [47]	2023	At least four ANC visits, childbirth at a health institution, and a PNC within 24 hours			
Mengistie H.M. et al [48]	2023	At least four ANC visits, delivery assisted by SBA and PNC within 48 hours			
Merga G et al [49]	2023	At least four ANC visits, childbirth aided by a skilled attendant, and at least one PNC within 6 weeks			

ANC: Antenatal care, SBA: Skilled birth attendants, PNC: Postnatal care.

* Since content of care were not considered in the studies, only the number of recommended ANC (ANC4+) and PNC visits (four) were considered for the comparison.

** Studies that included either institutional delivery, facility delivery, skilled birth attendants, or skilled birth attendants in health institutions in their definitions were considered in alignment with service recommendation during childbirth.

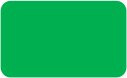
Consistent with the recommendation.

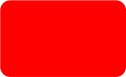
In consistent with the recommendation.

### Definition of “continuum of care” for maternal health care services

All articles defined the “maternal continuum of care” based on utilisation of maternal health care services across three periods: pregnancy, childbirth, and postpartum (Table 1).

### Pregnancy

Twenty studies expected four or more ANC visits to complete the continuum of care during pregnancy [21,23–26,28–30,32,33,35–37,42,43,45–49]. The remaining 9 studies required at least one ANC visit [22,27,31,34,38–41,44].

### Childbirth

In nine studies, giving birth at a health facility was a requirement to stay in the continuum of maternity care [25,29,32,34,39,41,44,45,47]. Of these, three studies emphasized that childbirth at the health facility should be attended by a skilled birth attendant [34,39,41]. Doctors, nurses, and midwives were listed as skilled birth attendants in two of these studies [39,41]. In fourteen of the included studies, the presence of a skilled birth attendant during childbirth, without mentioning the place of delivery, was used to define the continuum of care in this period [21,22,27,28,31,35–38,40,42,46,48,49]. Doctors, midwives, nurses, and public health officers were considered skilled birth attendants in four of the studies [35,38,40,46], and two studies added health extension workers to this list [22,31]. The terms institutional delivery or facility delivery and skilled birth attendant were used interchangeably in five studies [24,26,30,33,43].In the remaining study, childbirth at home or a health facility by a trained health professional was used to define the continuum of care during childbirth [23] (Table 1).

### Postpartum

The definition of the continuum of care during the postpartum period varied across studies. In ten studies, one postnatal care (PNC) visit within 42 days (6 weeks) was enough to complete the continuum of maternity care [22,25,30,31,38–41,44,49]. In most of these studies (n = 6), the place where the care was provided and the profession of the provider were not considered in the definitions [22,38,39,41,44,49]. In contrast, in the remaining four studies criteria were added: the care should be provided by a skilled health worker [30], the care should be provided by a health service provider at a health facility or home [40], at least once after discharge from health facilities or within the first week after childbirth at their home [25], and after discharge from the health facility within six weeks by skilled health personnel (medical doctors, midwives, nurses, health officers, or community health extension workers) or within the first week by community health extension workers during their home visit [31].

In nine studies, receiving a PNC within 24 hrs (n = 1) [47] and 48 hours (n = 8) [23,24,27,33,36,37,45,48] were requirements to complete the continuum of care in the postpartum period. Five studies included PNC in their definition without specifying the timing and/or number of visits: women should receive PNC after delivery (n = 3) [21,29,32], at least one PNC (n = 1) [26], and at least one PNC at a health facility or home (n = 1) [35].In two studies, four PNC visits were requirements to complete the continuum of care (the timing of the visits not specified) [42,43]. Furthermore, the remaining three studies defined the continuum of maternal healthcare during postpartum as follows, woman should have PNC within the first week after delivery at a health facility (excluding pre-discharge postnatal care) or home by a skilled provider [49], three PNC visits at a health facility or home, within 24 hours, within 3 days, and within a week [28], and a revisit to the facility to receive PNC after a facility birth [34].

### Alignment of the definitions with national maternal healthcare service recommendations

In twenty studies, the continuum of care during pregnancy was defined in line with the national recommendation of four or more ANC visits, while nine studies required fewer visits. During childbirth, all 29 studies included either institutional delivery, facility delivery, skilled birth attendants, or skilled birth attendants in health institutions in their definitions, indicating full alignment with national recommendations for this period. In addition, two studies, from the same authors, defined the continuum of care at PNC in line with the recommendations of four PNC contacts, whereas the remaining 27 studies required fewer contacts. Considering the national recommendations, only two studies, from the same authors, were in complete agreement with all the recommendations across the three periods: four or more ANC visits (using the former recommendation), institutional delivery/skilled birth attendants, and four PNC visits (Table 1).

## Discussion

This scoping review, to the best of our knowledge, is the first of its kind to examine the maternal continuum of care measurement metrics in Ethiopia. The measurement metrics were built based on the utilisation of health services across three periods, pregnancy, childbirth, and postpartum. The metrics focus on the number of contacts women should have, the place where these services should be provided, and the service provider's skill. None of the studies considered the quality of care provided during the visits. The number of contacts captures contact with the health system; however, it does not indicate the quality of services women receive. Evidence shows that maximizing coverage alone is insufficient to reduce maternal mortality [50]. Thus, continuum of care measurement metrics should consider the provision of recommended services during each contact and the women’s experience of care (interpersonal aspects of quality of care). This reflects the WHO framework for the quality of maternal and newborn health care [50], which defines quality of care as both the provision and the experience of care. Incorporating this framework into metric development can guide the design of measures that move beyond simple contact counts to capture the content of services delivered and women’s experience of care.

Developing a refined maternal continuum of care measurement metric is crucial for effectively monitoring maternal healthcare services and informing policymaking. A standardized metric will enable better tracking of progress and ensure that healthcare interventions align with national recommendations. Additionally, enhancing the monitoring of the maternal continuum of care with a more comprehensive metric can support targeted resource allocation and facilitate evidence-based policy reforms, ultimately fostering a more responsive and effective maternal healthcare system in Ethiopia.

We noted variability in the criteria used to define a continuum of care across the three periods. The postpartum period exhibited the most significant variation compared to the first two periods. Most maternal deaths in the country occur during this period [51–53], and the MOH recommends at least four postnatal contacts to prevent and manage maternal health conditions that contribute to mortality—within 24 hours, on day 3 (48–72 hours), between days 6 and 7, and at 6 weeks postpartum [10]. Yet, there is considerable inconsistency in how the continuum of care is defined. The two most commonly used definitions of the continuum of care during the postpartum period are “at least one postnatal care (PNC) visit within 42 days (6 weeks)” and “having a PNC visit within 48 hours”, respectively. Such simplified definitions overlook the clinical reasoning for multiple, timed contacts and limit the ability to track progress toward national targets. To address this, we recommend that the future measurement metric use a definition that specifies both the timing and number of postnatal contacts, aligned with national recommendations.

Timely service utilization is key to preventing maternal morbidity and mortality. Most maternal and neonatal deaths occur in the first 24 hrs of the postpartum period [54,55]. Early detection of conditions that may adversely affect the woman's and her newborn’s health and well-being is crucial to reduce mortality [10]. Although two studies defined the continuum of care in line with the four PNC contacts recommendation, the timing of the contacts was not considered.

Moreover, few studies (n = 2) defined the continuum of care in line with the national maternal healthcare service recommendations: four or more ANC visits, institutional delivery or skilled birth attendants, and four PNC visits [42,43]. Research plays a crucial role in supporting health systems by identifying challenges, providing solutions, assessing performance, and generating new knowledge. It also guides policy and decision-making [51]. While evidence on the status and effectiveness of the continuum of care is valuable for planning and monitoring maternal health programs, inconsistencies in measurements with maternal healthcare service recommendations limit the utilisation of research findings. Therefore, research should be designed and conducted to meet needs and ensure findings are practical.

Additionally, “Institutional delivery” and “skilled birth attendant (SBA)” are related but not the same, yet at times used interchangeably in the included articles. The former refers to giving birth in a health facility, such as a health center or hospital, which involves receiving professional care during childbirth [56]. However, SBAs are health professionals competent to provide effective and quality care during childbirth [57]. The 2019 Ethiopia Demographic and Health Survey (EDHS) national assessment shows a close but different result for the two services, 48% institutional delivery and 50% skilled birth attendant during childbirth [58]. The two terms are used interchangeably in government policy and strategy documents as well, which might contribute to the different definitions of the continuum of care during childbirth. MOH and partners working on maternal health should ensure healthcare recommendations are written clearly and consistently in their policy and strategy documents.

This review has the following limitations. Although English is the primary language used to report research findings in Ethiopia, articles written in other languages might be missed. The review did not include measurement metrics found in gray literature. Moreover, the quality of the included studies was not assessed.

## Conclusion

Current maternal continuum of care measurement metrics primarily focus on the coverage of maternal healthcare services. Moreover, most existing metrics are inconsistent with national maternal healthcare service recommendations. Our findings indicate a need for a consistent, holistic measurement metric that addresses both coverage and quality of the services while aligning with maternal health care service recommendations. By introducing a new standardized metric, efforts to improve the continuum of care will be strengthened with the necessary data to track progress and guide evidence-based interventions, ensuring a more integrated and effective maternal healthcare system. The development of this metric should leverage existing frameworks for assessing the quality of maternal healthcare services.

## Supporting information

S1 Checklist Completed Preferred Reporting Items for Systematic Reviews and Meta-Analyses extension for Scoping Reviews (PRISMA-ScR) checklist. From: Tricco AC, Lillie E, Zarin W, O'Brien KK, Colquhoun H, Levac D, et al. PRISMA Extension for Scoping Reviews (PRISMAScR): Checklist and Explanation. Ann Intern Med. 2018;169:467–473. https://doi.org/10.7326/M18-0850. Reproduced under the terms of the Creative Commons Attribution 4.0 International License (https://creativecommons.org/licenses/by/4.0/).
(PDF)

S1 Text Search strategies (search terms and results). (DOCX)
