# Supplementary material for: Assessing metrics for evaluating the maternal continuum of care in Ethiopia: A scoping review
Source: PLOS Glob Public Health. 2026 Sep 15;6(9):e0007303. doi: 10.1371/journal.pgph.0007303 (PMC13577583; doi:10.1371/journal.pgph.0007303)
Supplement: Supplementary file 2 [file pgph.0007303.s002.docx]

# **Search strategies**

Search date: October 27 to 29, 2023

**Step 01:** An initial search on MEDLINE (via PubMed) and Scopus databases.

PubMed: “Continuum of maternity care in Ethiopia”

Search result: 38 articles

Scopus: “Continuum AND Maternity AND Care AND Ethiopia”

Search result: 29 articles

**Step 02**: Search using all identified keywords and index terms across all included databases.

**PubMed**

(("Continuity of Patient Care"[Mesh] OR "Continuum" [tiab] OR "Continuum of Care" [tiab] OR "Care Continuum" [tiab] OR Continu*[tiab]) AND ("Maternal Health Services"[Mesh] OR "Maternal Health service" [tiab] OR "Maternity care" [tiab] OR "Maternity servic*"[tiab] OR "Obstetrics servic*"[tiab])) AND ("Ethiopia"[Mesh] or Ethiopia [tiab])

Search result: 182 articles

**Scopus**

TITLE-ABS-KEY ( "Continuum" OR "Continuum of Care" OR "Care Continuum" OR "Continuity of Care" OR "Care Continuity" OR continu* ) AND TITLE-ABS-KEY ( "Maternal Health Servic*" OR "Maternity care" OR "Maternity servic*" OR "Obstetrics servic*" ) AND TITLE-ABS-KEY ( ethiopia ) )

Search result: 112 articles

**Web of science**

((AB=(“Continuum” OR “Continuum of Care” OR “Care Continuum” OR “Continuity of Care” OR “Care Continuity” OR Continu*)) AND AB=( "Maternal Health Servic*" OR “Maternity care” OR “Maternity servic*” OR “Obstetrics servic*”)) AND AB=(Ethiopia)

Search result: 53 articles

**Google scholar**

allintitle: "Continuum" AND "maternity care" AND "Ethiopia"

allintitle: "Continuum" AND "maternity" AND "Ethiopia"

allintitle: "Continuum of care" AND "maternity" AND "Ethiopia"

allintitle: "Care Continuum " AND "maternity" AND "Ethiopia"

allintitle: "Continuum" AND "maternal health services" AND "Ethiopia"

allintitle: "Continuum of care" AND "maternal health services" AND "Ethiopia"

allintitle: "Continuity" AND "maternal health services" AND "Ethiopia"

allintitle: "Continuity of care " AND "maternal health services" AND "Ethiopia"

Search result: 80 articles

**Step 03**: Checking the reference list of the selected articles.

Search result: 2 articles

**Total search results: 496 articles**
